# Molecular profiling of invertebrate glia

**DOI:** 10.1002/glia.24623

**Published:** 2024-10-16

**Authors:** Maria D. Purice, Inês Lago‐Baldaia, Vilaiwan M. Fernandes, Aakanksha Singhvi

**Affiliations:** ^1^ Division of Basic Sciences Fred Hutchinson Cancer Center Seattle Washington USA; ^2^ Department of Biological Structure School of Medicine, University of Washington Seattle Washington USA; ^3^ Department of Cell and Developmental Biology University College London London UK

## Abstract

*Caenorhabditis elegans* and *Drosophila melanogaster* are powerful experimental models for uncovering fundamental tenets of nervous system organization and function. Findings over the last two decades show that molecular and cellular features are broadly conserved between invertebrates and vertebrates, indicating that insights derived from invertebrate models can broadly inform our understanding of glial operating principles across diverse species. In recent years, these model systems have led to exciting discoveries in glial biology and mechanisms of glia–neuron interactions. Here, we summarize studies that have applied current state‐of‐the‐art “‐omics” techniques to *C. elegans* and *D. melanogaster* glia. Coupled with the remarkable acceleration in the pace of mechanistic studies of glia biology in recent years, these indicate that invertebrate glia also exhibit striking molecular complexity, specificity, and heterogeneity. We provide an overview of these studies and discuss their implications as well as emerging questions where *C. elegans* and *D. melanogaster* are well‐poised to fill critical knowledge gaps in our understanding of glial biology.

## Main Points

- Summary of all ‐omic studies in *C. elegans* and *Drosophila*
- Assessment of insights, caveats, and future directions

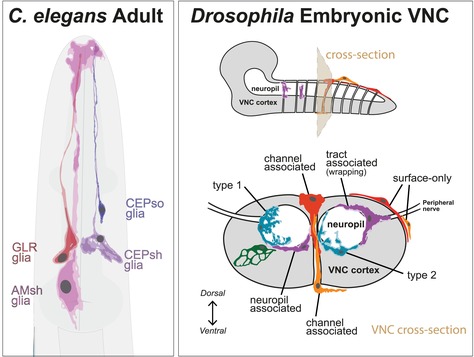

## 1 INTRODUCTION

Glia are evolutionarily conserved cell types with common features and functions across divergent species. They are posited to have coevolved in the urbilaterian ancestor along with cephalization and interneuron formation (Heiman & Shaham, 2007; Verkhratsky et al., 2019). In bilaterians, glial cells are critical to nervous system development and function, with roles in generating neurons and promoting neurite formation, to animal behavior and longevity.

The nervous systems of invertebrate model organisms, like *Caenorhabditis elegans* and *Drosophila melanogaster*, contain glia that contact and interact with neurons. Given their genetic tractability and molecular toolkits that allow cell resolution studies, it is not surprising that recent studies in these experimental models are yielding rapid mechanistic insight into glial roles in both the developing and mature nervous system. These studies are being further accelerated by the “‐omics” revolution. Here, we first describe the glial classification system of each model. We then survey the different molecular profiling studies that have been conducted thus far on *C. elegans* and *D. melanogaster* glia, including whole cell or nuclear RNA sequencing (RNA‐seq), ribosomal RNA profiling, metabolomics, and so on. We summarize the molecular insights they have yielded, as well as caveats for these studies. We conclude with an exploration of the emergent questions that these model organisms, in our view, are uniquely poised to address in glia biology.

As the current landscape of glial profiling explodes with data, we believe that the tractability of *C. elegans* and *D. melanogaster* allow for emerging ‐omics datasets to be validated in vivo with unparalleled speed and cellular precision. Thus, these models are well‐poised to provide molecular and mechanistic insights into glial biology.

## 2 INVERTEBRATE GLIAL CLASSIFICATION SYSTEMS

This section summarizes glial development and the classification systems for glia in both animal models, to contextualize profiling studies.

### 2.1 Caenorhabditis elegans

**FIGURE 1:**
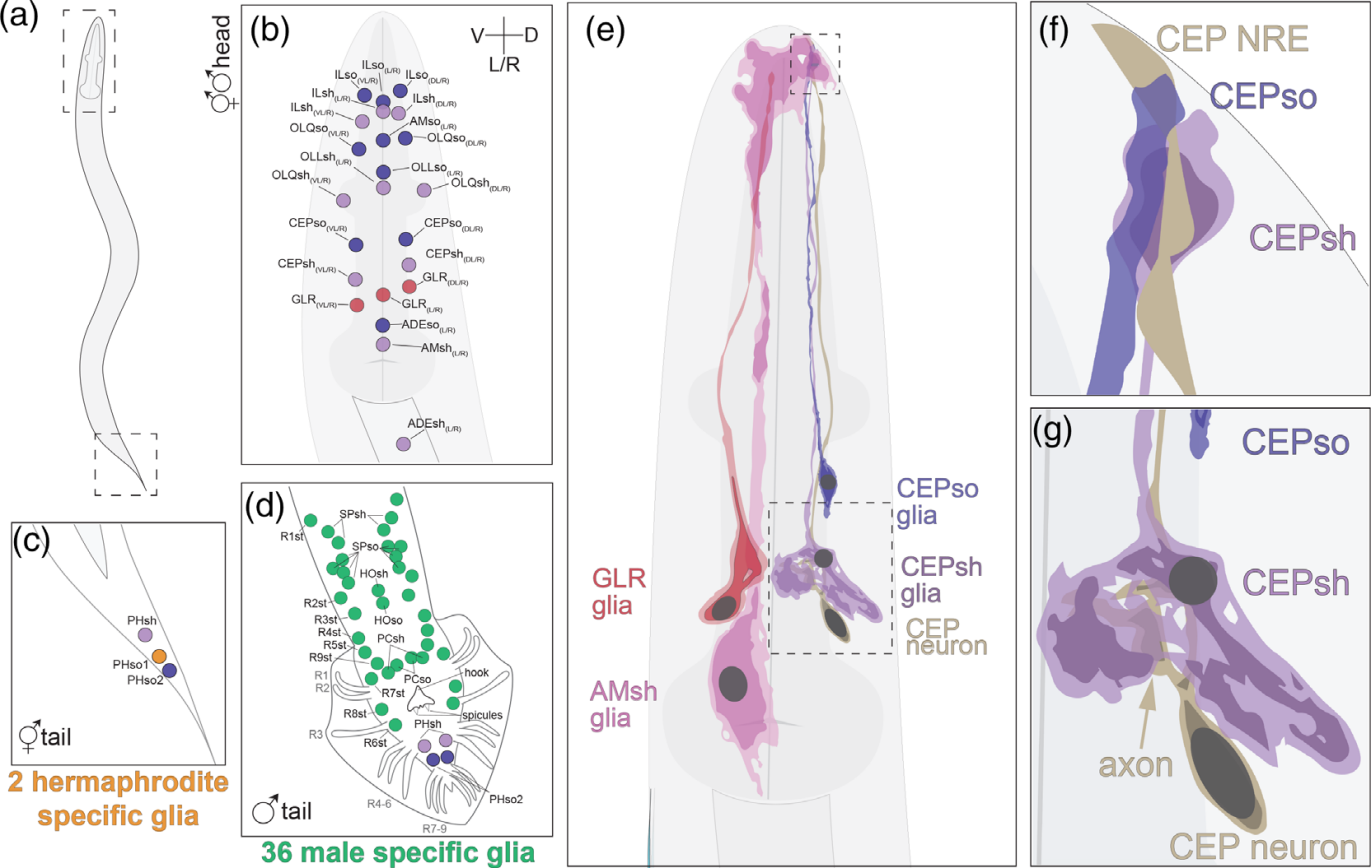
Anatomical and molecular characterization of adult *C. elegans* glial nuclei in hermaphrodites and males. (a) Schematic representation of an adult hermaphrodite worm with outlines of the head and tail regions. Close‐up of the head (b), hermaphrodite tail (c), and male tail (d). (e) Schematic examples of one mesoderm‐derived GLR glia, one AMsh glia, and one cephalic sense organ. Each hermaphrodite cephalic organ consists of one CEP sensory neuron (brown) that associates with CEPso glia (blue) and CEPsh glia (purple). (f) Close‐up of the nose tip (e top box) showing direct interactions between the CEP neuronal receptive ending, the CEPso glia, and CEPsh glia. (g) Close up of the nerve ring area (e lower box) showing interactions between the CEP neuron axons and CEPsh glial sheath, but no physical interaction with the CEPso glia in this region. Cells in schematics e–g were traced from fluorescent images and the color was changed in Adobe Illustrator. Shading within each cell indicates fluorescence intensity of original images. Adapted from Purice et al. (2023).

The adult *C. elegans* nervous system is composed of 302 neurons and 56 glia in the hermaphrodite (somatically female), and 387 neurons and 82 glial cells in the male, some of which are multinucleated (Molina‐García et al., 2020; Sammut et al., 2015; Sulston & Horvitz, 1977; Sulston et al., 1983) (Figure 1A–D). Each neuron and glial cell is lineally invariant, meaning that their developmental lineage trajectory from the single‐cell zygote to the mature individual cell is the same across animals (Sulston & Horvitz, 1977; Ward et al., 1975). Further, each glia–neuron contact is also cellularly invariant across animals (Sulston & Horvitz, 1977; Ward et al., 1975). Finally, the adult neural connectome has been mapped in both sexes, the first for a multicellular animal nervous system (Doroquez et al., 2014; Jarrell et al., 2012; Varshney et al., 2011; Ward et al., 1975; White et al., 1986). The animal uses this cellularly hardwired but behaviorally plastic neural network for multiple distinct (and experimentally quantifiable) behaviors, including sleep, locomotion, mating, sensory perception, and learning and memory (Bargmann, 1998; Kaletsky et al., 2018; Packer et al., 2019; White et al., 1986; Yemini et al., 2021). The cell biology, properties, and function of neurons underlying these are reasonably well characterized using genetic and ‐omic techniques. Together, these features showcase this organism as an exceptional model for investigating the contributions of glial cells to neural circuit functions and animal behaviors (Oikonomou & Shaham, 2011; Raiders et al., 2021; Ray & Singhvi, 2021; Singhvi & Shaham, 2019; Singhvi et al., 2024).

Of the 56 total glia in the adult hermaphrodite, 50 glial cells are of neuroectodermal origin like vertebrate macroglia (astrocytes, oligodendrocytes, PNS glia) (Figure 1B). Six glial cells, called GLR glia, are of mesodermal origin like vertebrate microglia, mid/hindbrain pericytes, and endothelia (Ginhoux et al., 2010; Shaham, 2006; Sulston & Horvitz, 1977; Sulston et al., 1983) (Figure 1B, E). All neuroectoderm‐derived glia in *C. elegans* associate with sensory neurons in sense organs, whose name is where their cell ID nomenclature derives from (Figure 1F). There are seven sex‐shared bilateral sense organs, comprising 16 glial cell types and totalling 48 glia (Figure 1B–D). The organs are the bilateral amphids (AM) and phasmids (PH), fourfold cephalic (CEP), pseudo‐hexaradiate (six‐fold) symmetry outer labial (OL) and inner labial (IL) (Ward et al., 1975) and two anterior and posterior deirids (AD, PD), that reside in the animal midbody (Ward et al., 1975). Each sex‐shared sense organ contains two neuroectodermal glial cell types, the sheath (sh) and socket (so) glia (Figure 1E,F). All neuroectoderm‐ and mesoderm‐lineage glia originate embryonically, except for sex‐shared phasmid socket (PHso1, PHso2) glia, which are born in the L1‐L2 larval stage, and male‐specific glia that are born in the L2‐L4 larval stages.

Both sh and so glial cells extend processes that fasciculate with neurons at the animal's periphery, as well as each other (Figure 1F). Sheath glia make cell:cell junctions with neurons and socket glia, while socket glia do so with sheath and overlying epithelia (Figure 1F). Based on sense‐organ and modality sensed, dendrite‐endings of cognate sensory neurons either traverse a channel made jointly by sheath and socket glia to sample the external environment or embed through the channel into the cuticle (Figure 1F), or are enwrapped by sheath glia. In addition to this anatomy of the anterior glial ending, the four cephalic sheath (CEPsh) glia also extend posterior processes to distally ensheath the synapse‐rich circular nerve ring, which is the animal's brain neuropil (Figure 1G). Finally, the six‐fold symmetric mesodermal GLR glia enwrap this neuropil proximally (Figure 1E), suggesting that these together may seal the nerve ring like a blood–brain barrier (Altun & Hall, 2009; Oikonomou & Shaham, 2011; Singhvi & Shaham, 2019; Stefanakis et al., 2024; White et al., 1986). GLR glia also make gap junction with at least one neuron (RME), and head muscles (White et al., 1986).

Given its invariant lineages, *C. elegans* is also an ideal setting to probe cellular sex differences in the nervous system. Five glia are sexually dimorphic by anatomy: the CEPsh, cephalic socket (CEPso), amphid socket (AMso), phasmid socket 1 (PHso1), and PHso2. These suggest precise setting to compare glial sexual dimorphism at single‐cell resolution. Briefly, AMso divide only in the male to give rise to the MCM interneurons (Sammut et al., 2015). CEPsh and CEPso glia interact with male‐specific chemosensory CEM neurons only in the male (Sulston et al., 1980). And lastly, PHso1 glia transdifferentiate (and a small percentage divide) into PHD neurons only in the male and are therefore lost in this sex (Molina‐García et al., 2020; Sammut et al., 2015) (Figure 1D). Additionally, while present in both sexes, PHso2 functions anatomically as the male phasmid socket, analogous to hermaphrodite PHso1 (Hirsh et al., 1976; Sulston et al., 1980). Males also have an additional 28 ectodermal glia which reside in the tail, an organ used for mating (Sulston et al., 1980) (Figure 1D). These categorize into seven anatomical groups contained within four male‐specific sense organs in the male tail. These are either unilateral [hook (HO)], or bilateral [spicule (SP); postcloacal (PC), rays (R)]. HO, SP, and PC have sheath and socket glia, while each ray has a single glial cell that may have both sheath and socket attributes (Lints & Hall, 2009). In addition, the PC and HO sense organs also have associated epithelial cells (Lints & Hall, 2009).

The anatomy of *C. elegans* glia across sexes already suggests that glia across the animal or between sexes may not be equivalent. There is also emerging evidence for functional heterogeneity in specific glia–neuron interactions. Recent studies have shown that at least some *C. elegans* glia impact neural development (neurite specification, neurogenesis, and process guidance), neuron properties and circuit function (regulating neuronal ionic milieu and activity, engulfing neuron fragments, tri‐partite synapse modulation, and neurotransmitter diffusion), animal behaviors (sensory responses, mating, sleep, and locomotion), organismal lifespan, and stress response. Readers are directed to other reviews for comprehensive summaries of these glial functions (see Purice et al., 2024; Shaham, 2015; Singhvi & Shaham, 2019; Singhvi et al., 2024). Dissection of the underlying mechanisms has revealed molecular players that are conserved and expressed in glia of other species. Thus, *C. elegans* glia are molecularly and functionally diverse, and study of their heterogeneity and sexual dimorphism in this powerful genetic model will likely be broadly relevant across species.

### 2.2 *Drosophila* *melanogaster*

*Drosophila* glia share key morphological and functional characteristics with their vertebrate counterparts. They maintain neurotransmitter and ionic balance, provide trophic support for neurons, perform immune functions, and modulate neural circuitry (Freeman, 2015; Lago‐Baldaia et al., 2020; Yildirim et al., 2019). In *Drosophila*, glia were initially estimated to constitute ~10% of the ~200,000 cells in the adult brain (Konstantinides et al., 2018; Raji & Potter, 2021), although a recent quantification places them at a much higher proportion of ~40% (Jiao et al., 2022). Most glia originate from the neuroectoderm and are generated by neural stem cells (called neuroblasts), which produce both neurons and glia, or from dedicated glial progenitors called glioblasts (Jones, 2001). Several distinct modes of gliogenesis have been documented in *Drosophila*. Of these, gliogenesis from neuroblast lineages undergoing temporal patterning most closely resembles gliogenesis in the vertebrate retina and cortex (Chen et al., 2016; Chotard et al., 2005; Kohwi & Doe, 2013; Li et al., 2013). In these lineages, diverse neuron types are generated over the lifetime of a given neuroblast, with developmental time acting as an axis for cell type diversification. Intriguingly, in both *Drosophila* and vertebrates, these lineages tend to produce glia in late or terminal temporal windows after neurogenesis (Kohwi & Doe, 2013; Konstantinides et al., 2022; Li et al., 2013).

**FIGURE 2:**
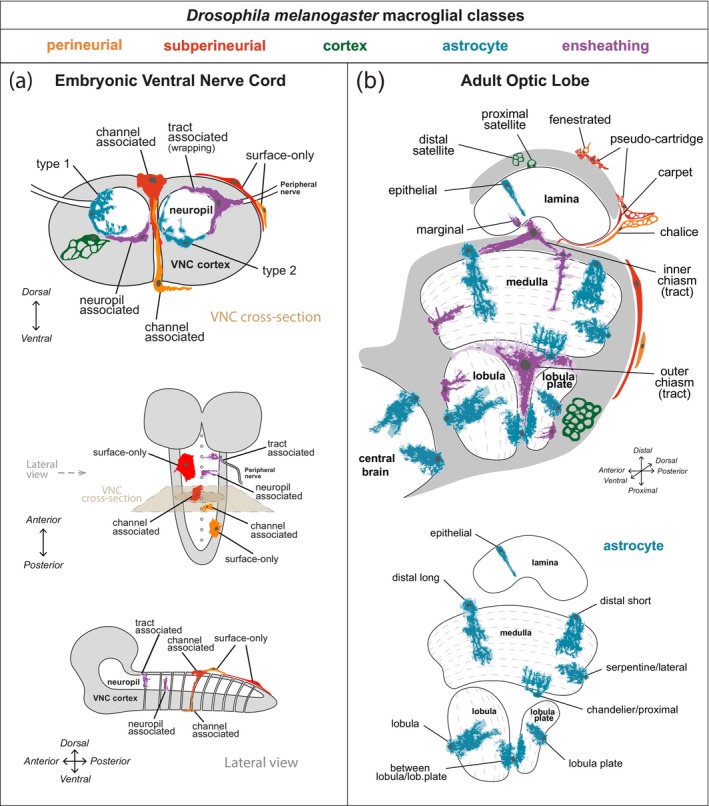
Schematics of *Drosophila melanogaster* macroglial classes and morphologies in the embryonic ventral nerve cord and adult optic lobes. The five macroglial classes are color‐coded as follows: perineurial glia (orange), subperieurial glia (red), cortex glia (green), astrocytes (blue), and ensheathing glia (purple). The neuropils are white and the cortex is grey. (a) Schematics showing the stereotypical morphologies of the embryonic VNC glial classes in (a‐top) cross‐sectional view, (a‐middle) surface view, and (a‐bottom) lateral view. Depending on the glial class, morphologies vary discretely (e.g., perineurial glia) or along a continuum (e.g., subperineurial glia; two extremes of subperineurial morphology are depicted). (b) A cross‐sectional view of the adult optic lobe showing the four neuropils (neuropil layering indicated by dashed lines). Distinct glial morphologies are observed for each of the classes, exemplified for the astrocytes, which exhibit 8 distinct morphologies distributed across the four neuropils such that they vary by volume, primary branch number and the neuropil layers they occupy. Adapted from Lago‐Baldaia et al. (Lago‐Baldaia et al., 2023)

*Drosophila* glia are organized into five classes of macroglia, all of which are found across embryonic, larval, and adult stages (Figure 2A,B). They all express the pan‐glial marker reversed polarity (*repo*), which encodes a homeodomain transcription factor important for glial identity and differentiation (Halter et al., 1995; Yuasa et al., 2003). These classes are defined by morphology, cell body position, and association with specific anatomical structures: the outer and inner surface glia, cortex glia, ensheathing glia, and astrocytes (Figure 2A,B). In the central nervous system (CNS), neuropils house synaptic connections, while neuronal cell bodies are positioned at the neuropil periphery (called the cortex) (Figure 2A,B; grey). Surface glia comprise two sheet‐like glial classes known as perineurial and subperineurial glia (or outer and inner surface glia). Together, they form a double‐layered surface that spans the nervous system, acting as a blood (hemolymph)‐brain barrier (BBB) (Figure 2A,B). Cortex glia surround neuronal cell bodies in the cortical regions of the CNS and loosely resemble mammalian satellite glia of the peripheral nervous system (PNS). Ensheathing glia can be further subdivided into two subclasses based on morphology (Figure 2A,B). Tract ensheathing glia (also referred to as wrapping glia in the embryo, and chiasm glia in the optic lobe) ensheath axon tracts between neuropils, or nerve bundles in the PNS and are thought to be analogous to nonmyelinating Remak Schwann cells, whereas neuropil ensheathing glia encase neuropil borders (Figure 2A,B). *Drosophila* astrocytes extend numerous fine projections into the neuropil to associate with neuronal synapses, similar to their vertebrate counterparts (Figure 2A,B). As in vertebrates, *Drosophila* glia exhibit morphological diversity both across and within glial classes (Edwards & Meinertzhagen, 2010; Hartenstein, 2011; Kremer et al., 2017; Lago‐Baldaia et al., 2022; Richier et al., 2017; Salazar et al., 2022). The latter is perhaps best exemplified in the adult *Drosophila* visual system where eight distinct morphological categories of astrocytes can be defined based on branching, and the neuropil and neuropil subdomains they occupy (Figure 2B) (Lago‐Baldaia et al., 2023; Richier et al., 2017). For example, epithelial glia are the only astrocytes found in the lamina neuropil and are morphologically and transcriptionally distinct from astrocytes of the other visual system neuropils. In contrast, chandelier glia are a morphological category of medulla neuropil astrocytes found on the proximal surface of the medulla neuropil. Although they differ in volume and branching from astrocytes found along the distal and lateral medulla, to date they are transcriptionally indistinguishable (Lago‐Baldaia et al., 2023).

Although the adult *Drosophila* brain does not contain glia of mesodermal origin, like microglia in vertebrates or GLR in *C. elegans*, immune‐related functions famously attributed to microglia, such as phagocytosis of neuronal corpses and debris clearance, and so on, have been reported for all five *Drosophila* macroglial classes (Doherty et al., 2009; Kurant et al., 2008; Neukomm et al., 2014; Tasdemir‐Yilmaz & Freeman, 2014). Unlike the adult stages, cells originating from the mesoderm are found in the *Drosophila* embryonic brain: (1) Hemocytes are macrophages that originate from the head mesoderm and migrate along the midline of the ventral nerve cord (VNC) before dispersing laterally throughout the embryo (Tepass et al., 1994). During these stages, hemocytes phagocytose apoptotic corpses, including neuronal corpses in the brain prior to glial maturation and formation of the surface glial brain barrier (Evans et al., 2010). (2) Midline glia are ensheathing cells positioned along the VNC midline and are thought to be analogous to the vertebrate floorplate because of their contribution to boundary and guidance functions (Jacobs, 2000; Klämbt et al., 1996). These cells do not express the pan‐glial marker *repo* (Giesen et al., 1997) and are later eliminated during the larval and pupal stages (Awad & Truman, 1997; Sonnenfeld & Jacobs, 1994). In this review, we will limit our discussion to the five classes of macroglia.

## 3 OVERVIEW OF PROFILING APPROACHES: *C. elegans* AND *D. melanogaster*

*Caenorhabditis elegans* was the first multicellular organism to have its genome sequenced (*C. elegans* Sequencing Consortium, 1998), followed by *Drosophila*, a little over a decade later (Adams et al., 2000). Both models have served as invaluable testing grounds for emerging sequencing approaches as they have evolved. These have included applications of microarrays, bulk RNA‐sequencing (RNA‐seq), whole cell or nuclear RNA‐seq of isolated cell types, translating ribosome affinity purification followed by RNA sequencing (TRAP‐seq), DNA adenine methyltransferase identification (DamID), a chromatin profiling approach, and assays for transposase‐accessible chromatin with sequencing (ATAC‐Seq) (see Table 1).

**TABLE 1:** Comprehensive summary of ‐omics studies on glial cells in *C. elegans* and *Drosophila*.

“‐Omics” approach	Technique	Species	Cell/tissue type	Genetic line/reagent	Timepoint	References
Transcriptomics	Microarray	*C. elegans*	AMsh glia	*vap1::GFP*	Cultured dissociated embryonic cells	Bacaj et al. (2008)
		*D. melanogaster*	Surface glia	*9‐137‐Gal4*	Adult	DeSalvo et al. (2014)
	Bulk whole cell RNA‐seq	*C. elegans*	AMsh glia	*T02B11.3::PGM87*	Cultured dissociated embryonic cells	Grant et al. (2015)
		*C. elegans*	AMsh glia	*F16F9.3::DsRed*	Larva (L3‐L4 stage)	Wallace et al. (2016)
		*C. elegans*	CEPsh glia	*hlh‐17::GFP*	Larva (L2‐L3 stage)	Katz et al. (2019)
		*C. elegans*	GLR glia	*nep‐2prom7::nls::yfp*	Larva (L4 stage)	Stefanakis et al. (2024)
		*D. melanogaster*	Midline cells	*3.7sim‐Gal4*	Embryo	Fontana and Crews (2012)
		*D. melanogaster*	Astrocytes	*alrm‐Gal4*	3rd instar larva and adult	Huang et al. (2015)
		*D. melanogaster*	Astrocytes	*alrm‐Gal4*	Adult	Ng et al. (2016)
		*D. melanogaster*	VNC		Adult (with and without peripheral nerve injury)	Purice et al. (2017)
		*D. melanogaster*	Eye‐antennal imaginal disc		Late 2nd, mid 3rd and late 3rd instar larva	Torres‐Oliva et al. (2018)
		*D. melanogaster*	Glia and neurons	*repo‐Gal4* and *elav‐Gal4*	3rd instar larva	Pindyurin et al. (2018)
		*D. melanogaster*	Carpet glia	*C135‐Gal4*	Adult	Ho et al. (2019)
		*D. melanogaster*	Epithelial glia	*R37D04‐p65ADZp + R55A03‐ZpGdbd*	Adult	Davis et al. (2020)
				*R37D04‐p65ADZp + R42B05‐ZpGdbd*		
				*R42B05‐p65ADZp + R55A03‐ZpGdbd*		
			Marginal glia	*R35E04‐p65ADZp + R65B12‐ZpGdbd*		
			Proximal satellite glia	*R38A07‐p65ADZp + R87B09‐ZpGdbd*		
				*R42B05‐p65ADZp + R87B09‐ZpGdbd*		
		*D. melanogaster*	Glia and neurons	*repo‐Gal4/elav‐Gal4*	Adult	Özel et al. (2020)
		*D. melanogaster*	Glia and neurons	*repo‐Gal4/elav‐Gal4*	Adult	Simões et al. (2022)
		*D. melanogaster*	Glia and neurons	*repo‐Gal4/nSyb‐Gal4*	Adult	Ichinose et al. (2023)
	Bulk TRAP‐Seq	*D. melanogaster*	Astrocytes	*alrm‐Gal4*	3rd instar larva and adult	Huang et al. (2015)
		*D. melanogaster*	Astrocytes	*alrm‐Gal4*	Adult	Ng et al. (2016)
		*D. melanogaster*	Astrocytes	*eaat1‐Gal4*	Adult	You et al. (2021)
	sn/scRNA‐seq	*C. elegans*	Whole embryo (including glia)		Larva (L2 stage)—hermaphrodite	Cao et al. (2017)
		*C. elegans*	Whole larvae (including glia)		Embryo—hermaphrodite	Packer et al. (2019)
		*C. elegans*	Neurons (including glia)	*several neuronal subgroups*	Larva (L4 stage)—hermaphrodite	Taylor et al. (2021)
		*C. elegans*	All neuroectoderm glia (glia‐specific)	*mir‐228:nls::RFP*	Adult (Day 1)—hermaphrodite and male	Purice et al. (2023)
		*C. elegans*	Whole animal (including glia)		Adult—hermaphrodite (Days 1, 3, 5, 8, 11, and 15)	Roux et al. (2023)
		*C. elegans*	Whole animal (including glia)		Adult (Day 1)—hermaphrodite	Ghaddar et al. (2023)
		*D. melanogaster*	Brain (midbrain + optic lobes)		Adult (0, 1, 3, 6, 9, 15, 30, and 50 days old)	Davie et al. (2018)
		*D. melanogaster*	Midbrain		Adult	Croset et al. (2018)
		*D. melanogaster*	Brain and VNC		1st instar larva	Brunet Avalos et al. (2019)
		*D. melanogaster*	Brain and VNC		3rd instar larva	Ravenscroft et al. (2020)
		*D. melanogaster*	Eye‐antennal imaginal disc		3rd instar larva	Bravo González‐Blas et al. (2020)
		*D. melanogaster*	Optic lobe		Pupa (0, 12, 24, 36, 48, 60, 72, 84, and 96 h APF)	Özel et al. (2020)
		*D. melanogaster*	Optic lobe		Pupa (15, 30, 40, 50, and 70 h APF) and adult	Kurmangaliyev et al. (2020)
		*D. melanogaster*	VNC		Adult	Allen et al. (2020)
		*D. melanogaster*	Optic lobe		3rd instar larva and P15 pupa	Konstantinides et al. (2022)
		*D. melanogaster*	Whole body (dissected tissues, including glia)		Adult	Li et al. (2022)
		*D. melanogaster*	Leg imaginal disc (including glia)		3rd instar larva	Tse et al. (2022)
		*D. melanogaster*	Brain		Adult	Park et al. (2022)
		*D. melanogaster*	Brain and/or VNC		1st and 2nd instar larva (1, 24, and 48 h after hatching)	Corrales et al. (2022)
		*D. melanogaster*	Brain		Adult (1, 10, and 20 days old)	Wu et al. (2023)
		*D. melanogaster*	Whole body (including glia)		Embryo (stage 17)	Lago‐Baldaia et al. (2023)
		*D. melanogaster*	Midbrain		Adult	Dopp et al. (2024)
		*D. melanogaster*	VNC		3rd instar larva	Nguyen et al. (2024)
Chromatin Accessibility (and transcription)	Bulk ATAC‐Seq (& other)	*C. elegans*	Whole animal		Embryo, four larval stages, adult (Days 1, 2, 6, 9, and 13)	Jänes et al. (2018)
	Bulk ATAC‐Seq	*D. melanogaster*	Whole head, glia, neurons and clock neurons	*repo‐Gal4, nSyb‐Gal4* and *Clk856‐Gal4*	Adult	Ma, Ojha, et al. (2024) and Ma, Zhang, et al. (2024)
	sc‐ATAC‐Seq	*C. elegans*	Whole larvae (including glia)		Larva (L2 Stage)	Durham et al. (2021)
	sc‐ATAC‐Seq	*C. elegans*	Whole larvae (including glia)		Larva (L2 Stage)	Durham et al. (2021)
		*C. elegans*	Whole animal (including glia)		Embryo, larval Stage 3, young adult	Daugherty et al. (2017)
		*C. elegans*	Whole animal (including glia)		Embryos, arrested larval stage 1	Ho et al. (2017)
	scATAC‐Seq	*D. melanogaster*	Whole CNS		Adult	Davie et al. (2018)
		*D. melanogaster*	Whole CNS		3rd instar larva, Pupa (0, 3, 6, 12, 24, 48, and 72 h APF) and adult	Janssens et al. (2022)
	DamID/TaDa	*D. melanogaster*	Surface glia and Subperineurial glia only	*R54C07‐Gal4* and *R71C08‐Gal4, UAS‐Dam:Pol II, UAS‐Dam*	3rd instar larva	Contreras et al. (2021)
		*D. melanogaster*	Carpet glia	*C135‐Gal4, UAS‐Dam:Pol II, UAS‐Dam*	Adult	Ho et al. (2019)
TF binding	ChIP‐Seq	*C. elegans & D. melanogaster*	Whole animal	219 worm and 267 fly TF protein traps	Worm: Embryo, four larval stages, young adult	Kudron et al. (2018)
					Fly: Embryo, wandering larvae (W3L), white‐prepupae (WPP), pupa, adult, Kc167 cell line	
	CUT&Tag	*D. melanogaster*	Whole CNS	anti‐Repo	Adult	Janssens et al. (2022)
	DamID/TaDa	*C. elegans*	seam and hypodermal cells	*lin‐22:dam* and *nhr‐25:dam* under *wrt‐2*p	Larval stages 2 and 4	Katsanos et al. (2022)
		*C. elegans*	muscle and pharynx	*daf‐16:dam* under *hsp‐16.1*p	∼96 h after bleaching gravid adults	Schuster et al. (2010)
		*C. elegans*	AWC neurons and pharyngeal muscle	*daf‐16:dam* under *hsp‐16.41*p	Laval stage 2 and young adult	van de Walle (2020)
		*C. elegans*	muscle, intestine, and XXX cells	*myo‐3*, *egl‐5p* (6.5 kb), *elt‐2*, *sdf‐9* for Cre or cell‐specific FACS; *rpb‐6:dam* under *hsp‐16.2*p	1.5‐fold stage embryo and young adult	Gómez‐Saldivar et al. (2020)
Metabolomics	Mass spectrometry	*D. melanogaster*	Glia and neurons	*repo‐Gal4* and *nSyb‐Gal4*	Larva	Volkenhoff et al. (2015)
		*D. melanogaster*	Whole CNS		Larva	Bailey et al. (2015)
		*D. melanogaster*	Whole head		Adult	Li et al. (2023)

*Note*: This table presents a collection of research focused on the ‐omics analyses of *C. elegans* and *D. melanogaster* nervous systems. Datasets with no entries under “Genetic lines” were produced from dissected tissues or structures.

Early on, these studies relied on purified populations or subpopulations of individual glial cell types, taking advantage of the genetic access and availability of cell type‐specific enhancers and genetic toolkits. In both systems, these yielded data that led to additional molecular catalogs of genes expressed in glia and new molecular tools. In *C. elegans*, they also confirmed predicted glial functions, suggested previously unknown functions and molecular cues, and provided a basis for revealing cross‐species conservation of molecular signatures and functions (Bacaj et al., 2008; Grant et al., 2015). Similarly, microarray profiling of *Drosophila* surface glia highlighted chemical and metabolic barrier functions similar to that of the mammalian BBB (DeSalvo et al., 2014). These profiling studies generated cell‐specific glial signatures, that have aided annotation of single‐cell sequencing data (as described below).

The advent of microfluidic devices, designed to isolate individual cells or nuclei in droplets for parallel sequencing, marks the current era of widely applied RNA‐seq and ATAC‐seq to single cells or nuclei across various organisms, including *C. elegans* and *D. melanogaster*. Due to its small cell number, the *C. elegans* L2 larvae was the first multicellular organism to be entirely mapped by single‐cell transcriptome profiling via a combinatorial indexing strategy to capture the transcriptomes of individual cells (Cao et al., 2017). t‐distributed stochastic neighbor embedding (tSNE) plots derived from these whole animal scRNA‐seq datasets identified multiple glial cell clusters, the first indication of molecular heterogeneity across glial subtypes in the animal (Cao et al., 2017; Packer et al., 2019). Mining and in vivo analyses of these data unveiled novel cell‐type‐specific promoters for glia (Fung et al., 2020). Extending this method to profile glia by single‐nuclear RNA‐seq (snRNA‐seq), *C. elegans* has also become the first model with a complete molecular atlas of all neuroectoderm‐derived adult glia across the nervous system of both sexes (Purice et al., 2023).

Likewise in *Drosophila*, single‐cell RNA‐seq (scRNA‐seq) and scATAC‐seq datasets now exist for numerous organs including the brain (both larval and adult) (Brunet Avalos et al., 2019; Corrales et al., 2022; Davie et al., 2018; Dopp et al., 2024; Janssens et al., 2022; Ma, Ojha, et al., 2024; Nguyen et al., 2024; Park et al., 2022; Wu et al., 2023). Additionally, a community effort spearheaded by the Luo, Aerts, and Deplancke labs, constructed the Fly Cell Atlas (flycellatlas.org), a repository of publicly available snRNA‐seq and scATAC‐seq data of several adult fly organs (Li et al., 2022). For both worms and flies, sc/nRNA‐seq and scATAC‐seq has been extensively applied for studying the nervous system at different developmental stages as well as in the context of aging.

Using sc/nRNA‐seq provides an unbiased method to identify cell types and/or states in a tissue without prior knowledge. However, annotating specific cell types/state within these extensive datasets poses a significant challenge. Alongside labor‐intensive in vivo validation of gene expression, computational methods that transform cell type‐specific bulk RNA‐seq datasets, enabling their integration with scRNA‐seq datasets, have been developed and used to annotate neuronal and glial clusters across both worm and fly cell sc/nRNA‐seq datasets. Further, scRNA‐seq datasets have also been used to validate data generated using tandem affinity purification of intact nuclei (TAPIN) RNA‐seq (Davis et al., 2020).

## 4 MOLECULAR PROFILING OF *C. elegans* GLIA

Below is a summary of the different studies that profile *C. elegans* glia, organized by methods used and types of glia, as relevant. Whole‐animal ‐omics studies that include glial cells but do not evaluate glia biology are listed (Table 1) but not discussed further here.

### 4.1 Microarray profiling of individual glia

The AMsh glia (Figure 1E, pink) in the major sense organ of the animal (amphid) was the first glial cell to be molecularly profiled by oligonucleotide array sequencing. Ablation of the AMsh glia leads to deficits in morphology and/or function of the sensory neurons it physically associated with (Bacaj et al., 2008). To uncover glial factors controlling these neuronal properties, oligonucleotide gene array studies were performed on embryonic AMsh glia (Bacaj et al., 2008). This landmark study identified 298 enriched transcripts including those for FIG‐1/thrombospondin‐domain protein. Validations of this enrichment by expression studies and phenotypic characterization showed that FIG‐1 enriches in AMsh glia and regulates sensory neuron properties (Bacaj et al., 2008). This was a striking spotlight into cross‐species analogy, as mammalian astrocyte‐derived thrombospondins had been shown to regulate synaptogenesis (Cheng et al., 2016; Christopherson et al., 2005; Xu et al., 2010). Subsequently, another enriched AMsh glial transcript in this dataset, KCC‐3/K‐Cl cotransporter was also determined to be a glial regulator of associated AFD thermosensory neuron shape and thereby animal thermosensory behaviors (Ray et al., 2024; Singhvi et al., 2016; Tanis et al., 2009; Yoshida et al., 2016).

### 4.2 Bulk cell‐specific profiling of individual glia

Since then, four bulk RNA‐seq studies on individual glia at different developmental stages or conditions have been reported (Grant et al., 2015; Katz et al., 2019; Stefanakis et al., 2024; Wallace et al., 2016). These have profiled three glia (AMsh, CEPsh, and GLR glia [schematics of these glia shown in Figure 1E]), as summarized below.

#### 4.2.1 AMsh glia

Two studies performed bulk RNAseq analysis on AMsh glia isolated by fluorescent activated cell sorter (FACS) at different developmental stages. Like the microarray profiling, Grant et al. (2015) sorted cultured embryonic AMsh glia and identified 1135 AMsh‐enriched transcripts. This uncovered several anion channels and transporters enriched in AMsh glia, and this study validated in vivo the expression of CLH‐1/ClC chloride channels in AMsh glia by cell‐specific rescue experiments and expression pattern analyses. They also used genetic knockout studies to show that CLH‐1 regulates glial pH regulation and bicarbonate buffering (Grant et al., 2015).

Wallace et al. (2016) isolated AMsh glia from late‐stage *C. elegans* larvae and uncovered 598 AMsh glia‐enriched transcripts. To validate this data, they performed RNAi against 140 of these genes and screened adult animals for sensory deficits. This uncovered that RNAi against *pros‐1* results in similar defects to AMsh glia as AMsh ablation, including defective morphology of glia‐ensheathed sensory neuronal receptive endings (NREs) and defective neuronal functions and animal behaviors (Wallace et al., 2016). Interestingly, *pros‐1* encodes a Prospero‐related homeodomain transcription factor. While Prospero is classically known for determining cell fate decisions during nervous system development in *Drosophila* and mammals (Doe et al., 1991; Stergiopoulos et al., 2014), the authors showed that PROS‐1 has distinct postdevelopmental functions in the AMsh glia of adult animals in modulating neuronal properties and animal sensory behaviors. Finally, they showed that *pros‐1* regulates the AMsh glia secretome (Wallace et al., 2016).

Interestingly, both studies recovered only partial overlap of transcripts with the prior microarray study, even though all focused on a single, invariant glial cell, the AMsh. The Grant et al. (2015) study, which also isolated cultured embryonic AMsh glia, recovered 97/297 (32%) overlap in enriched transcripts, with 169 unchanged, and the remainder either depleted or not recovered. Similarly, Wallace et al. (2016) recovered 72/297 overlapping transcripts with the microarray study, although in this case the AMsh glia were collected from a different developmental stage and using a different reporter for cell‐sorting. It remains to be determined whether these disparities can be attributed to differences in transcript analysis (Grant et al., 2015) (microarray vs. RNA‐seq), statistical methods, or animal staging (embryo vs. larval). It will also be relevant to compare the two RNA‐seq profiles with each other and single‐cell profiles for AMsh glia (see below). Nonetheless, this variance in the transcriptome profile of a single invariant glial cell underlines the criticality in vivo validations before inferring biological insights from profiling datasets.

#### 4.2.2 CEPsh glia

Katz et al. (2019) performed RNA‐seq on larval FACS‐isolated CEPsh glia. Hierarchical clustering and rank ordering on CEPsh‐enriched genes compared with mouse homologs enriched in different brain cell types showed that CEPsh glia are most similar to murine astrocytes (Katz et al., 2019). This again validated cross‐species analogy of glial functions, since CEPsh glia ensheath multiple processes/synapses, contribute to at least one tripartite synapse, and were previously shown to have astrocyte‐like functions in neural development (Colón‐Ramos et al., 2007; Katz et al., 2018; Rapti et al., 2017; Yoshimura et al., 2008). In line with this, the profiling study showed that CEPsh glia express multiple transporters and receptors including the homolog of the astrocytic glutamate transporter GLT1/EAAT1/GLAST (Katz et al., 2019). By genetic mutant analyses, Katz et al. (2019) showed that loss of *glt‐1* leads to uncontrolled extracellular glutamate levels, which drives repetitive behavior in animals. This is striking as astrocyte glia across species, from *Drosophila* to mammals, regulate glutamate homeostasis through glutamate transporters GLAST/EAAT1 and GLT1/EAAT2 (Mahmoud et al., 2019; Matsuno et al., 2019; Rothstein et al., 1996). Thus, these insights revealed both functional and global molecular signature analogy of CEPsh glia with mammalian astrocytes.

#### 4.2.3 GLR glia

To profile the six mesoderm‐lineage GLR glia, Stefanakis et al. (2024) first identified a GLR‐specific transgenic reporter tool by promoter‐bashing. Using this, they performed bulk RNA‐seq on late larval FACS‐isolated GLR glia. This data identified two regulators of GLR glial development. Briefly, LET‐381/FoxF, promotes specification and maintenance of GLR cell fate, while UNC‐30/Pitx transcription factor controls GLR glia morphology and represses acquisition of alternative mesodermal fates (Stefanakis et al., 2024). Interestingly, gene ontology analysis revealed that GLR‐enriched genes are over‐represented in murine astrocytes as well as blood–brain barrier endothelial cells. The authors thus suggested that GLR glia likely execute merged astrocytic and endothelial functions, perhaps to account for limited glial cell numbers in the animal (Stefanakis et al., 2024).

### 4.3 Single‐cell profiling of *C. elegans* glia

*Caenorhabditis elegans* is the first model organism with a complete molecular atlas of neuroectoderm‐lineage glia of the entire animal nervous system, and across both sexes (wormglia.org) (Purice et al., 2023). Briefly, this study performed snRNA‐seq on FACS‐isolated adult glial nuclei of both sexes. Barring the few glia noted above, most glia in this animal have been poorly studied molecularly. So, the authors developed custom machine‐learning and analytical algorithms to uncover glia‐specific and glia‐subset molecular signatures. Finally, they validated many of the *in silico* finds in vivo, and uncovered molecular reporters for all *C. elegans* glia (Purice et al., 2023). In addition to this work, other studies have reported whole animal single‐cell profiling of all cells, across developmental and adult stages. While these also revealed multiple, putative glial clusters, only a few were identified due to a lack of prior insight into molecular markers for glia (Cao et al., 2017; Ghaddar et al., 2023; Packer et al., 2019; Roux et al., 2023; Taylor et al., 2021). The toolkits and molecular signatures generated by Purice et al. (2023) will enable mining of these existing datasets to dissect glial profiles across developmental stages at single‐cell resolution. It also provides molecular marker tools for deep sequencing by bulk cell‐specific ‐omics. Here, we summarize salient findings derived from these datasets.

#### 4.3.1 Glial heterogeneity

Socket glia associate with epithelial cells, and like them, secrete cuticle (Chisholm & Hsiao, 2012). Not surprisingly, then, transcription factor (TF) analysis of scRNA‐seq from L2 larval animals uncovered that epithelia‐associated TF‐encoding genes *blmp‐1*/*PRDM1* and *nhr‐25*/*Nr5a2* were also associated with gene expression in socket glia, excretory cells, and rectal cells, suggesting a role for these TFs in glial development (Cao et al., 2017). The roles of *blmp‐1*/*PRDM1* and *nhr‐25*/*Nr5a2* have not yet been explored in glial development in vertebrates, although these genes have been implicated in early embryogenesis in ectodermal cell fate specification and neural development, respectively (Prajapati et al., 2019; Stergiopoulos & Politis, 2016). Consistent with this, by using hierarchical clustering, a method that groups molecular profiles based on their levels of similarity, Purice et al. (2023) identified that *C. elegans* sheath and socket glia clustered based on functional relatedness (all sheath vs. all socket), rather than sense‐organ proximity (sheath/socket of one organ) or developmental lineage relatedness (Purice et al., 2023; Sulston et al., 1983). Taken together, these studies imply shared function between socket glia and epithelia. Further, by snRNA‐seq, *C. elegans* glia map to multiple clusters on the uniform manifold approximation and projection (UMAP), suggesting that they are molecularly heterogeneous (Purice et al., 2023). In addition, even glia with pseudo‐hexaradiate symmetry, such as ILso glia, segregate into distinct clusters, in both embryonic scRNA‐seq and adult snRNA‐seq datasets implying disparate molecular profiles (Packer et al., 2019; Purice et al., 2023). These findings track recent studies of their development, which occurs via convergent differentiation of three symmetric pairs of lineages that diverge in embryogenesis (Mizeracka & Heiman, 2015; Sulston et al., 1983). Thus, molecular insights from these datasets will be exciting to validate and define how anatomically identical glia exhibit different molecular profiles, and the functional implications thereof.

#### 4.3.2 Sexual dimorphism

Glial‐specific snRNA‐seq also reveal that anatomical and molecular sex dimorphism do not overlay. Some glia that are anatomically sexually dimorphic (AMso, CEPsh, CEPso, and PHso1) are not apparently different molecularly, while other anatomically identical glia exhibit sex‐dimorphism molecularly (i.e., PHsh) (Purice et al., 2023). It remains to be seen if this is because sex‐dimorphism is restricted to certain developmental critical periods and does not persist in adulthood, where the glial snRNA‐seq was derived; or if snRNAseq profiling depth is limiting for such analyses. Future studies will be required to tease these nuances apart and dissect the functional consequences of sexual dimorphism that was observed for some glia.

#### 4.3.3 Aging

*Caenorhabditis elegans* is an established model for organismal and neural aging studies (Arey & Murphy, 2017; Kenyon, 2010; Son et al., 2019). A whole animal single‐cell RNA‐seq dataset across six time points during animal age was recently reported, which reveals coordinated changes in metabolic, proteostasis, and stress‐response genes with cell type specificity (Roux et al., 2023). This study cataloged the most significantly upregulated/downregulated transcription factors and uncovered that seven out of eight top age‐regulated TFs are well‐known regulators of *C. elegans* longevity, including *skn‐1/Nrf2*. Interestingly, *skn‐1/Nrf2* showed cell type specificity with an increase in expression and activity in neurons and many other cell types with aging, but a drastic decrease in glial cells (Roux et al., 2023). This is intriguing because while NRF2 activation in age‐related neurodegenerative diseases occurs in glia, its mechanism of action in these cells remains unclear (Brackhan et al., 2023; Liddell, 2017). Further, this study found that unlike neurons, glial clusters did not upregulate cytosolic chaperones with age, and their ER resident proteins and ER stress‐response systems exhibited different aging profiles; suggesting that glia may have different cellular responses with aging (Roux et al., 2023). Barring these insights, most glial clusters in Roux et al. (2023) were unidentified, thus cell‐specific glial molecular aging remains unexplored in this animal. It will be exciting to mine these existing datasets to define how individual glia, now identifiable by studies of Purice and colleagues, track animal aging.

#### 4.3.4 Glial neuropeptide signaling

Glial neuropeptide signaling has been implicated in *C. elegans* organismal longevity (Frakes et al., 2020; Yin et al., 2017) and diverse neuropeptides expressed in glial cells have been recently revealed by sc/nRNA‐seq (Ghaddar et al., 2023; Purice et al., 2023; Roux et al., 2023; Taylor et al., 2021). The machinery that glia deploy to process and release neuropeptides is unexplored across species (Ubink et al., 2003). Strikingly, both snRNA‐seq and in vivo studies confirm that *C. elegans* glia do not express the same dense‐core vesicle processing or release machinery as neurons (Purice et al., 2023; Russo, 2017; Speese et al., 2007). This raises the question of how glia package and release biologically relevant neuropeptides, and if such glia‐specific machinery for neuropeptide signaling is conserved cross‐species.

## 5 MOLECULAR PROFILING OF *D. melanogaster* GLIA

Numerous bulk and single cell approaches have been applied to profile mRNAs or chromatin accessibility in the *Drosophila* brain or specifically across all glia, specific glial classes or subclasses, and at different developmental stages or during aging. These include metabolomics, microarray assays, TRAP‐seq, Targeted DamID, TAPIN RNA‐seq, Ribo‐seq, sc/nRNA‐seq, and scATAC‐seq, with at least one dataset available for every glial class and for several subclasses (Table 1). While still early days for exploring glial biology in *Drosophila* ‐omics datasets, these studies have identified key differences between glial and neuronal biology, and new molecular markers to better identify and study individual glial classes/subclasses. They have seeded further exploration into glial roles in processes, such as metabolite transport, neurotransmitter recycling and sleep, have shed light on glial diversity, and reinforced the notion that glial functions are evolutionarily conserved from invertebrate to vertebrates. Below we summarize some of these insights as well as the caveats associated with these studies.

### 5.1 Insights into pan‐glial versus pan‐neuronal biology gained from bulk profiling applications

In *Drosophila*, various “bulk” approaches have been employed to profile gene expression, metabolism, and lipids in the nervous system. In many instances, these approaches have been applied broadly to the CNS, or to all glia or neurons. Still, when combined with cell type‐specific genetic perturbations, bulk ‐omics approaches have provided valuable insights into distinctions between glial and neuronal biology and glia–neuron interactions, summarized below.

#### 5.1.1 Lactate shuttling from glia to neurons

In vertebrates, astrocytes support the metabolic needs of neurons by releasing lactate that neurons then metabolize, a process called the astrocyte‐neuron lactate shuttle. In a landmark study, Volkenhoff and colleagues showed that glycolysis‐driven lactate shuttling from glia to neurons also occurs in *Drosophila* (Volkenhoff et al., 2015). Unlike vertebrates, which use glucose as the primary energy‐supplying metabolite, insects like *Drosophila* rely on trehalose (Chatterjee & Perrimon, 2021). Volkenhoff et al. (2015) demonstrated that trehalose transporters (Tret[s]) and glycolytic enzymes are required in glia but not in neurons. In a powerful application of bulk metabolomics, the authors performed high‐performance liquid chromatography—mass spectrometry (HPLC‐MS) on glial and neuronal populations isolated by FACS, which were cultured with isotope‐labeled trehalose. Their analysis revealed that glia (but not neurons) secrete alanine and lactate. Furthermore, they showed that neurons could uptake alanine and lactate for respiration *in vivo* (Volkenhoff et al., 2015). In summary, their work underscored the evolutionarily conserved nature of glial metabolic support for neurons. Consistent with these findings, Ichinose and colleagues found that, compared to glia, neurons showed increased translation of transcripts associated with oxidative phosphorylation and mitochondrial ribosomal proteins, whereas glia showed increased translation of glycolysis‐related genes compared with neurons (Ichinose et al., 2023).

#### 5.1.2 Protective functions of lipid droplets in glia

Lipid droplets (LDs) are intracellular organelles that store neutral lipids, such as cholesteryl esters and triacylglycerols (Islimye et al., 2022). They are observed predominantly in glia, ependymal cells and microglia in the mammalian nervous system, where they accumulate under conditions of redox stress associated with high levels of reactive oxygen species (ROS) (Islimye et al., 2022). LDs can play a variety of context‐dependent roles. Indeed, depending on the biological context glial‐LDs have been ascribed with either harmful or protective roles (Islimye et al., 2022). Under hypoxic conditions LDs accumulate in subperineurial and cortex glia of the developing CNS, where they play a protective role to support continued neuroblast (*Drosophila* neural stem cells) proliferation (Bailey et al., 2015). By combining cell‐specific genetic manipulations with lipidomics (tandem mass spectrometry and gas chromatography—mass spectrometry) and multi‐isotope imaging mass spectrometry, Bailey et al. (2015) showed that ROS increase in the CNS under hypoxic conditions and induce LD accumulation in glia. This strategy diverts polyunsaturated fatty acids away from membranes to sequester them into glial LDs, where they are less vulnerable to peroxidation (Bailey et al., 2015). In this way, glial LDs protect neuroblast proteins from damage due to ROS‐induced peroxidation (Bailey et al., 2015; Li et al., 2018). In a similar vein, other studies have found that glial LDs may also sequester already peroxidated lipids as a means of neuroprotection (Ioannou et al., 2019; Liu et al., 2015). In related work, Liu et al. (2017) linked glia–neuron lactate shuttling and LD formation under conditions of redox stress in both flies and mammals (Liu et al., 2017). They showed that mitochondrial dysfunction in *Drosophila* photoreceptors leads to increased ROS and LD accumulation in glia‐like pigment cells in the *Drosophila* compound eye (Liu et al., 2017). Interestingly, glia–neuron lactate shuttling is needed for neurons to metabolize lactate to AcCoA, which in turn stimulates neuronal lipogenesis (Liu et al., 2017). These fatty acids then become peroxidated due to high ROS and are exported from neurons into glia where they are trafficked into glial‐LDs. Mammalian astrocyte‐neuron lactate shuttling was also shown to be critical for ROS‐Induced astrocyte‐LD accumulation (Liu et al., 2017).

#### 5.1.3 Distinct glial and neuronal responses to injury

Glial cells exhibit fast and dramatic reactions to neural injury, yet the transcriptional programs that direct these complex innate immune responses remain unclear. Purice et al. (2017) explored molecular responses of glial cells to neuronal injury using a novel *Drosophila* injury model that induces widespread glial responses in the adult VNC, offering a new platform to study glial responses akin to those observed in the *Drosophila* CNS (Doherty et al., 2009; Logan et al., 2012; Tasdemir‐Yilmaz & Freeman, 2014; Ziegenfuss et al., 2008) and mammalian systems (Chung et al., 2013, 2015; Herrmann et al., 2008; Kim et al., 2002; Konishi et al., 2022; Liddelow & Barres, 2017; Yu et al., 1995). Bulk RNA‐seq on whole VNCs post axotomy revealed a diverse array of responsive signaling pathways, highlighting matrix metalloproteinase‐1 (MMP‐1) as a key player (Purice et al., 2017). MMP‐1, acutely induced in ensheathing glia upon axonal injury, requires the well‐conserved engulfment receptor Draper/MEGF‐10 and downstream AP‐1 and STAT92E signaling. When glia are depleted of MMP‐1, they fail to infiltrate the neuropil regions properly or clear the degenerating axonal debris, establishing MMP‐1 as a critical player in mediating effective glial responses to neural damage (Purice et al., 2017).

In a recent study, Simões et al. (2022) used bulk transcriptomics as a starting point to investigate how scattered quiescent neural progenitors are recruited to sites of local brain injury. By performing bulk RNA‐seq on injured and intact adult fly brains, the authors found that most genes differentially expressed between the two groups encoded proteins that associated with the extracellular space or were involved in immune and stress responses. Among the injury‐induced upregulated genes was *swim*, which encodes a lipocalin‐like protein that facilitates extracellular transport of hydrophobic molecules, including Wnt (Simões et al., 2022). An endogenous Swim protein‐trap revealed that its expression was induced specifically in glia upon injury, a result confirmed by bulk RNA‐seq of FACS‐glia versus neurons isolated from intact and injured brains. Subsequently, the authors demonstrated that ensheathing glia and neurons in the vicinity of the injury site respond by secreting Swim and Wingless/Wnt, respectively. Thus, Swim, produced by glia, promotes the dispersion of Wg/Wnt, produced by neurons, to drive proliferation of dormant neural progenitors scattered throughout the brain (Simões et al., 2022).

### 5.2 Bulk profiling approaches applied to specific glial classes or subclasses

As in *C. elegans*, cell‐type‐specific bulk profiling approaches are also commonly deployed in *Drosophila*. Most rely on isolating specific cell populations, typically labeled by the Gal4/UAS system (e.g., through FACS). As well, Targeted DNA adenine methyltransferase identification (TaDa) is a relatively simple and versatile method for profiling gene expression, chromatin accessibility and for identifying the genome‐wide binding sites of chromatin‐associated proteins in vivo, without the need for cell isolation (Southall et al., 2013). In particular, polymerase‐II TaDa identifies loci bound by Pol‐II within the window of Pol‐II‐DNA adenine methyltransferase expression, and therefore identifies genes that were actively transcribed during the same period. Such bulk approaches are particularly useful for profiling rare cell types for which driver tools exist. For example, carpet glia are an extremely rare optic lobe subperineurial glial type. With only 4 carpet glia per animal, this cell type has been impossible to profile by sc/nRNA‐seq but instead has been investigated successfully using TaDa (Ho et al., 2019).

The primary caveat associated with bulk approaches pertains to the purity of the input cell population, which influences the specificity and accuracy of the output data. Consequently, in vivo validation is crucial to ensure the reliability of findings. Therefore, as with most ‐omics applications, bulk profiling approaches are most valuable when employed as part of a multipronged strategy to address a given question.

#### 5.2.1 Metabolite transport and nutrient sensing by surface glia

Of the two surface glial classes, perineurial glia are smaller and more numerous than subperineural glia with cell ratios of 3:1 (Kremer et al., 2017). Together they form a permeability barrier between the brain and hemolymph, similar to vertebrate blood–brain barrier endothelial cells. Like BBB endothelia tight junctions, subperineurial glia form the functionally analogous septate junctions with each other.

To better understand the function of surface glia, DeSalvo et al. (2014) used microarrays to profile the gene expression of FACS purified surface glia of the adult brain. Since subperineurial glia are polypoid and less abundant than perineurial glia, a caveat of this study relates to the unknown relative contribution of each of the surface glial classes to the final dataset. This study identified several genes enriched in surface glia relative to all glia (labeled by *repo‐Gal4*) and to all neurons (labeled by the pan‐neuronal driver, *elav‐Gal4*), many of which have been validated in vivo or by other profiling techniques (Allen et al., 2020; Konstantinides et al., 2018; McMullen et al., 2020; Parkhurst et al., 2018). These included genes related to nutrient and metabolite transport (e.g., fly homologs of solute carrier [SLC] transporters), drug metabolism and efflux for neuronal protection (e.g., fly homologs of ABC [ATP binding cassette] transporters), and cell adhesion (e.g., septate junction components). Mammalian BBB endothelial cells express genes associated with the same molecular functions (reviewed in Kadry et al., 2020). For example, the vesicular monoamine transporter, *Vmat*, predicted homolog of *SLC18A1/VMAT1*, was one of the most highly expressed genes enriched in surface glia based on the microarray data (DeSalvo et al., 2014). Moreover, the authors showed that the glia‐expressed variant VMAT‐B is expressed by perineurial glia only, consistent with a prior report that it was expressed by fenestrated glia, a perineurial glial subclass specific to the lamina neuropil of the optic lobe (Romero‐Calderón et al., 2008).

Not surprisingly given their expression profile, surface glia also play important roles in nutrient sensing. They were shown to mediate neural stem cell reactivation after a state of quiescence in response to larval feeding (Britton & Edgar, 1998; Chell & Brand, 2010; Sousa‐Nunes et al., 2011). Interestingly, scRNA‐seq of the early larval VNC under conditions of nutrient deprivation showed that compared with fed animals, glia from starved animals, including surface glia, underwent a shift in their fatty acid metabolism by upregulating genes involved in fatty acid catabolism, among others, while downregulating genes, such as *fatty acid synthase 1* (Brunet Avalos et al., 2019). To further define nutrient responsive gene expression in surface glia Contreras et al. (2021) applied polymerase‐II TaDa to surface glia (both perineurial and subperineurial glia, and subperineurial glia only) in larva subjected to normal feeding conditions and to nutrient restriction. They identified scarface (*scaf*), which encodes a secreted serine protease, as a nutrient responsive gene, whose expression decreased under nutrient restriction (Contreras et al., 2021). Notably, *scaf* was also detected in the DeSalvo et al. (2014) microarray dataset. Under normal feeding, knockdown of *scaf* in subperineurial glia led to decreased neural stem cell proliferation, increased perineurial glia proliferation and increased subperineurial glia endoreplication, suggesting that Scaf reigns‐in growth and proliferation of surface glia, to ensure a balance that is required to maintain neural stem cell proliferation (Contreras et al., 2021).

Beyond more obvious roles of supplying metabolites for nervous system energetics, surface glia, in their roles as gatekeepers, have also emerged as important regulators of sleep. Sleep was found to enhance endocytosis in surface glia (Artiushin et al., 2018). Moreover, disrupting endocytosis in surface glia was sufficient to increase sleep (Artiushin et al., 2018). Li et al. (2023) performed LC–MS metabolomic analysis on flies with increased sleep caused by pan‐glial inhibition of endocytosis and found that acylcarnitines accumulate in the heads of these animals. Endocytosis can have both direct and indirect effects on the levels of membrane‐associated transporters by affecting endocytosis‐dependent trafficking and/or recycling; therefore, to identify glial transporters that regulate sleep, Li et al. (2023) complimented their metabolomic analyses with surface glial knockdowns against transporters and receptors informed by the DeSalvo et al. (2014) microarray dataset. They identified a handful of transporters and trafficking factors that affected sleep, including *Vmat*. Perineurial glia‐specific knockdown of *Vmat* increased sleep, consistent with previous reports of that *Vmat* mutants exhibit increased sleep (Nall & Sehgal, 2013). As well, knocking down the lipid transporters *LRP1* together with *megalin*/*LRP2* (which were also identified by DeSalvo et al., 2014) in perineurial glia specifically led to increased sleep and associated accumulation of acylcarnitines (Li et al., 2023). Thus, both metabolomics and transcriptomics have played an important role in shaping our understanding of metabolite transport by surface glia into the brain, highlighting that transport of specific metabolite categories by particular glial classes can regulate defined processes and behaviors, such as sleep.

#### 5.2.2 Defining the molecular functions of astrocytes

Huang et al. (2015), Ng et al. (2016), and You et al. (2021) employed TRAP‐seq to characterize larval and adult astrocytes in *Drosophila*. TRAP‐seq profiles mRNAs specifically associated with ribosomes and are thus more likely to reflect active translation than nuclear or whole cell mRNA profiling. Briefly, the Gal4/UAS system is used to express the large ribosomal subunit (L10a) tagged with GFP in a target cell population (Thomas et al., 2012). Earlier studies used *astrocytic leucine‐rich repeat molecule (alrm)‐Gal4* to drive expression of tagged ribosomes (Huang et al., 2015; Ng et al., 2016), whereas more recently, You et al. (2021), also included *Excitatory amino acid transporter 1 (Eaat1)‐Gal4* as a driver. While *alrm‐Gal4* is commonly referred to as an astrocyte‐specific driver, it also drives expression in tract ensheathing glia of the adult optic lobe, called chiasm glia; whereas *Eaat1* drives expression in both astrocytes and neuropil ensheathing glia in adults (Krzeptowski et al., 2018; Lago‐Baldaia et al., 2023). Therefore, by focusing on genes common to *alrm‐Gal4* and *Eaat1‐Gal4* TRAP‐seq results, a more accurate astrocyte‐specific translatome could be obtained (You et al., 2021).

Consistent with known gene expression patterns, these datasets identified genes involved in neurotransmitter recycling and homeostasis (i.e., *ebony* and *Dopamine acetyltransferase*) and glutamate signaling (i.e., *Glutamine synthetase 2* and *Eaat1*) (Davla et al., 2020; Freeman et al., 2003; Kato et al., 2020; MacNamee et al., 2016; Stork et al., 2012; Suh & Jackson, 2007). In addition, Gene Ontology enrichment analysis revealed enrichment of genes associated with metabolism, energy production, translation, vesicle‐mediated transport and secretion, and chromatin remodeling (Huang et al., 2015; Ng et al., 2016). Interestingly, genes associated with vesicle‐mediated transport and chromatin remodeling were enriched specifically in adult but not larval astrocytes (Huang et al., 2015), hinting at additional biological functions of mature astrocytes. Importantly, in support of evolutionarily conserved functions between *Drosophila* and mammalian astrocytes, the *alrm‐Gal4* TRAP‐seq dataset showed a strong overlap with genes expressed by mouse astrocytes but not mouse oligodendrocytes (Huang et al., 2015).

In a more recent study, You et al. used TRAP‐seq to identify astrocytic genes with cyclic ribosomal association patterns in animals initially entrained by day/night cycles followed by free‐running in darkness (You et al., 2021). Their dataset identified not only genes that were previously identified as having rhythmic circadian expression profiles but also several novel genes with apparently rhythmic translation; of note, stress and innate immune response‐related genes were overrepresented (You et al., 2021).

Collectively, these studies highlight evolutionarily conserved functions between insect and mammalian glial types. Moreover, they serve as proof of concept, demonstrating their potential to serve as a foundational framework for future exploratory investigations.

### 5.3 Single cell profiling approaches

Sc/nRNA‐seq provides an unbiased method to identify cell types and/or states in a tissue without prior knowledge. However, annotating specific cell types/states within these extensive datasets is labor intensive and poses a significant challenge. Despite the recent abundance of sc/nRNA‐seq datasets available for the *Drosophila* nervous system, glia have been largely overlooked. Indeed, glial clusters are often left unannotated or minimally annotated to the level of the five broad classes and often lack in vivo validation (Allen et al., 2020; Bravo González‐Blas et al., 2020; Brunet Avalos et al., 2019; Corrales et al., 2022; Croset et al., 2018; Davie et al., 2018; Dopp et al., 2024; Konstantinides et al., 2022; Kurmangaliyev et al., 2020; Nguyen et al., 2024; Özel et al., 2021; Park et al., 2022; Ravenscroft et al., 2020; Tse et al., 2022; Wu et al., 2023).

#### 5.3.1 Glial diversity

Konstantinides et al. (2018) used drop‐seq, an early iteration of scRNA‐seq technology, to profile cells of the adult *Drosophila* optic lobe. Compared with present day scRNA‐seq technology, drop‐seq profiled fewer cells and to a shallower sequencing depth. Nonetheless, this dataset contained glial clusters that could be annotated to the level of the five main glial classes, but also included a cluster, which through in vivo validation of cluster markers was shown to correspond to the chiasm glia, an optic lobe‐specific tract ensheathing glia (Konstantinides et al., 2018) (Figure 2B). More recently, 10X Genomics sequencing technology was used to profile cells in the optic lobes of not only the adult, but also several developmental timepoints during the pupal stages and as early as the third instar larva (Kurmangaliyev et al., 2020; Özel et al., 2021). With increased cell numbers and improved sequencing depth, these independent datasets resolved 20 unique optic lobe glial transcriptional clusters in the adult and, though unannotated, hinted at potential glial subclass diversity. Similarly, Corrales et al. (2022) conducted scRNA‐seq on the entire CNS at three developmental time points in the larva and their clustering analyses produced distinct transcriptional clusters within the astrocyte, ensheathing, and cortex glial classes (Corrales et al., 2022). As cautioned above, without in vivo validation, it is impossible to draw conclusions about the meaning of within‐class transcriptional diversity in scRNA‐seq datasets.

Two groups have taken advantage of published datasets to create *Drosophila* glial cell atlases (Ferreira & Desplan, 2023; Lago‐Baldaia et al., 2023). Interestingly, as part of their initial data quality control and analyses, Lago‐Baldaia et al. (2023). Determined that some of the glial clusters expressed pan‐neuronal markers (*Rdl*, *Frq1*, and *Nckx30C*), which in vivo were confirmed to be restricted to neurons. Given the close interaction between glia and neurons throughout life, the authors posited that these cells represent either glial cells contaminated by neuronal transcripts or neuronal cells contaminated by glial transcripts, and therefore excluded them from further analyses (Lago‐Baldaia et al., 2023). Even after excluding contaminated cells and following standard quality control pipelines, Lago‐Baldaia et al. (2023) found that cluster analysis appeared to split apart cells belonging to the same cell type into distinct transcriptional clusters, due to stress signatures. Indeed, validating the expression pattern of marker transcripts mapped these clusters to the same cells in vivo (Lago‐Baldaia et al., 2023). If these interpretations hold true, comparing single‐cell to single‐nuclei RNA‐seq may be informative, as snRNA‐seq data of the same tissue would not be expected to contain such neuronal contamination artefacts. Another possible interpretation of the identity of some transcriptional clusters was proposed by Ferreira and Desplan (2023), who also analyzed glial clusters from a published scRNA‐seq dataset (Özel et al., 2021). Ferreira and Desplan showed that the standard tissue dissociation protocols used to prepare cells for isolation in droplets broke apart large glial cells into multiple fragments, which were captured in individual droplets and sequenced as individual “cells.” They argue that for at least some glial cell types, some transcriptional clusters in UMAP space represent glial cell somas while other clusters represent glial processes. Nonetheless, both studies provide caution with regards to extrapolating cell type diversity from cluster analyses without in vivo marker validation.

Lago‐Baldaia et al. (2023), who focused on embryonic glia and adult optic lobe glia, went on to systematically investigate the relationship between glial morphological and transcriptional diversity. The authors used clonal analysis to first validate previous reports of within‐class glial morphological diversity, and, in the process, identified new morphological categories (Lago‐Baldaia et al., 2023). They complemented these data by analyzing scRNA‐seq data of glia in the embryonic VNC and adult optic lobes (the latter from already published datasets), and by performing extensive in vivo validation of markers associated with different transcriptional clusters, as a means to map glial transcriptional signatures onto defined morphological categories. They observed two types of morphological variation: In the first, a continuum of morphologies was found between two extremes, which was the case for the embryonic subperineurial glia and ensheathing glia. In the second, discrete categories of stereotyped morphologies could be assigned easily, example for the case of the embryonic and adult optic lobe astrocytes, which could be categorized into two (Figure 2A) and eight (Figure 2B) distinct morphological categories, respectively. Nonetheless, irrespective of the type of morphological variation, detected transcriptional diversity did not align with morphological diversity (Lago‐Baldaia et al., 2023). Although Lago‐Baldaia et al. found extensive within‐class transcriptional diversity (predominantly for adult optic lobe glia, but also embryonic perineurial glia), unexpectedly, they found that glia exhibited more morphological diversity than detectable transcriptional diversity (Lago‐Baldaia et al., 2023).

Most within‐class transcriptional diversity in the adult optic lobe dataset could be attributed to glia associated with the lamina neuropil. Intriguingly, despite the lamina representing the simplest of all neuropils (in terms of neuronal diversity), no other neuropil exhibited within‐class transcriptional diversity despite extensive within‐class morphological diversity. This suggests that the transcriptional specialization observed in the lamina is unlikely to be due to increased circuit complexity. Differential gene expression analysis revealed that genes associated with immune‐related functions were upregulated in lamina glia compared to other optic lobe glia (Lago‐Baldaia et al., 2023). Lago‐Baldaia et al. argued that the lamina may be subject to more pathogenic insults than other optic lobe neuropils, due to its superficial position in the visual system, and thus that glial specialization may be driven primarily by adaptations to combat environmental insults (Lago‐Baldaia et al., 2023). It will be important to evaluate the generalizability of these findings in other systems.

Intriguingly, despite differences in developmental stage and origin, integrating the embryonic and adult glial datasets revealed that most embryonic glia coclustered by class with “general” adult optic lobe glia (i.e., medulla, lobula, and lobula plate glial classes but not lamina glia), highlighting a remarkable convergence in glial transcriptional profiles (Lago‐Baldaia et al., 2023). Glia associated with the lamina were transcriptionally distinct and did not co‐cluster with embryonic glia (Lago‐Baldaia et al., 2023). A notable exception was the chalice glia, a specialized transcriptionally distinct subclass of perineurial glia found at the margins of the lamina cortex, which coclustered with the embryonic channel perineurial glia, a specialized transcriptionally distinct subclass of embryonic perineurial glia associated with neuropil channels in the embryonic VNC (Lago‐Baldaia et al., 2023). Whether differences between embryonic channel perineurial glia and surface‐only perineurial glia are also driven by immune‐related genes, similar to lamina glia compared with other optic lobe glia, remains to be explored.

A major caveat when interpreting the results of this study is the limited depth of sequencing offered by scRNA‐seq technologies. It is conceivable that scRNA‐seq may fail to detect transcriptional diversity driven by genes expressed at low levels. However, the glial datasets were extracted from full optic lobe datasets, in which the same sequencing depth was sufficient to resolve over 100 neuronal transcriptional clusters, which aligned well with neuronal morphological categories when validated in vivo. Thus, if these datasets and analyses do indeed miss glial transcriptional diversity, it would imply that potential within‐class transcriptional differences fall well below the threshold that resolves most neuron types. It is also worth noting that the glial transcriptional diversity defined by Lago‐Baldaia et al. (2023) aligns closely with that predicted by glial (Gal4) enhancers identified from the Janelia GAL4 collection (Edwards & Meinertzhagen, 2010; Jenett et al., 2012; Kremer et al., 2017; Pfeiffer et al., 2008). Another caveat is that the optic lobe data analyzed in this study focused on mature glia in adults, leaving open the possibility that developmental transcriptional differences associated with lineage may be relevant to establishing distinct morphologies but that once established, glial transcriptional profiles converge (see section on Lineage relationships below). While unique transcription factor codes have been found to define specific neuron types or neurons from specific lineages, to date, no evidence of lineage‐specific transcription factor codes have been reported for astrocytes (Allen et al., 2020). A study exploring the development of astrocytes derived from motorneuron lineages found that astrocytes from the same lineage acquired diverse morphologies and did not express unique transcription factor codes according to lineage or morphology, as is the case for neurons (Enriquez et al., 2018). Overall, the data from Lago‐Baldaia et al. (2023) contend that for glia morphological diversity and neuropil complexity should not be presumed to correlate with transcriptional diversity.

#### 5.3.2 Lineage relationships between ensheathing glia and astrocytes

Whereas the developmental origin and lineage relationships for different glial classes in the embryonic VNC are well‐characterized, less is known for the larval and pupal stages. The only glial classes reported to be lineage‐related are the ensheathing glia and the astrocyte (Kato et al., 2020; Ren et al., 2018). In the embryonic VNC ensheathing glia and astrocytes arise from a common progenitor, as a consequence of a Notch‐mediated binary cell fate decision (Peco et al., 2016). In the central brain two distinct sources of progenitors (one originating during larval development and one during pupal development) again generate both ensheathing glia and astrocytes (Kato et al., 2020). Finally, the specialized ensheathing and astrocyte glial types of the lamina in the optic lobes (marginal and epithelial glia, respectively), which arise during the late larval and early pupal stages, also arise from a common progenitor (Chen et al., 2016; Dearborn & Kunes, 2004; Perez & Steller, 1996). This relationship appears universal as developmental scRNA‐seq of the optic lobes, spanning the late larval and pupal stages, also confirms a common origin for the epithelial and marginal glia of the lamina and paints a similar picture for ensheathing glia and astrocytes of the medulla, lobula and lobula plate neuropils (Ferreira & Desplan, 2023). Indeed, developmental scRNA‐seq datasets integrated through time suggests that cortex, surface, and chiasm glia are not lineage related whereas ensheathing glia and astrocytes appear to originate from the same pool (Ferreira & Desplan, 2023). These data also suggest two progenitor pools, one for the medulla and another for the lobula and lobula plate neuropils, consistent with the fact that neuroblasts, which generate the medulla neuropil neurons, ensheathing glia and astrocytes, arise from a neuroepithelium called the outer proliferation center; whereas a distinct neuroepithelium called the inner proliferation center, gives rise to the lobula and lobula plate neuropils and their associated ensheathing glia and astrocytes. Several studies have highlighted the many functional similarities between mammalian and *Drosophila* astrocytes (Freeman, 2015; Freeman & Doherty, 2006; Lago‐Baldaia et al., 2020; Yildirim et al., 2019), and many parallels are also apparent between mammalian oligodendrocytes and *Drosophila* ensheathing glia (Freeman & Doherty, 2006; Lago‐Baldaia et al., 2020; Yildirim et al., 2019). In mammals, depending on the brain region and environmental cues, oligodendrocyte precursor cells are capable of generating oligodendrocytes as well as astrocytes (Behar, 2001; Klum et al., 2018; Ma, Zhang, et al., 2024; Suzuki et al., 2017; Tatsumi et al., 2008; Zhao et al., 2009), thus suggesting that the lineage relationships between these glial classes may be conserved evolutionarily.

## 6 FUTURE PERSPECTIVE

The sheer volume and complexity of data being generated spanning multiomics techniques is advantageous for expanding our understanding of glial biology, but also brings additional challenges on how to analyze these data. The future of ‐omics research will have to take into consideration (1) standardization of current ‐omics technologies protocols, storage and sharing; (2) usage of advanced machine learning techniques to decipher complex datasets and aid in the discovery of new insights, and (3) integration of ‐omics data sets (discussed below). As ‐omics research in *C. elegans* and *Drosophila* expand toward proteomics and metabolomics in addition to the current transcriptomic approaches used, there will be a need to develop integrated analysis pipelines to merge data from multiple ‐omics techniques. Lastly, while ‐omics data can provide valuable insights, validation of findings in vivo is also crucial. *C. elegans* and *Drosophila* are powerful genetic model systems, which have genetic toolboxes that allow for rapid in vivo validation, making them ideal organisms for bridging the gap between ‐omics data and in vivo biology. We discuss below our view on the state of the field for each model organism and conclude with a perspective on how insights from both species may be synergistically integrated.

### 6.1 Drosophila

In *Drosophila*, sc/nRNA‐seq datasets are being generated at a rate that outpaces their systematic analysis, including in the context of glial biology. These rich, publicly available datasets open numerous avenues for exploring glial biology in the context of development, function, sex‐specific differences, aging, and disease. As with most large high‐throughput datasets, in vivo validation and accurate cluster annotation is a challenge and bottleneck. We anticipate that recent *Drosophila* glial atlases (Ferreira & Desplan, 2023; Lago‐Baldaia et al., 2023) will aid in this effort and serve as a valuable reference for other datasets.

Numerous transcriptomic and chromatin accessibility datasets are available across developmental stages and for distinct brain regions (summarized in Table 1). These offer an extensive and largely untapped resource for glial biologists. For *Drosophila*, while within‐class glial diversity has been explored in detail for the embryo and adult optic lobes (Lago‐Baldaia et al., 2023), it will be interesting to investigate whether other brain regions show similar trends; for example, do glia of the central brain also display more morphological diversity than transcriptional diversity? Are immune‐related genes the main driver of within‐class glial diversity? What are the gene regulatory networks that set and maintain these cell fates? Moreover, by comparing glial classes and subclasses across brain regions, we can address which genes and molecular functions are universal for a given glial class and which ones may be driven by brain region.

Data from mammalian models suggest that micro‐ and macroglia exhibit strong sex‐specific differences in the context of development and disease (Bobotis et al., 2023; Chowen & Garcia‐Segura, 2021; Forger et al., 2016; Rurak et al., 2022). Sex‐specific glial differences have already been documented in *Drosophila*, for example glia contribute to a larger proportion of male brains compared with female brains (42% vs. 37%, respectively) (Jiao et al., 2022). In *Drosophila*, a cell's sex is determined cell‐autonomously based on each cell's chromosome composition (XX vs. XY), which lead to activation of distinct branches of the sex‐determination pathway (Janzer & Steinmann‐Zwicky, 2001; Steinmann‐Zwicky et al., 1989). This peculiarity enables the “feminization” of male tissues or vice versa through cell‐autonomous manipulations of the sex determination pathway. Intriguingly, feminizing subperineurial glia in males disrupted their courtship behavior, which was phenocopied by disrupting specific G‐protein coupled receptors in the same cells (Hoxha et al., 2013). Distinct sex‐specific glial responses were also observed in the context of injury—whole animal heads were transcriptionally profiled over a time‐course following traumatic brain injury and revealed that female flies upregulated expression of the pan‐glial marker *repo* faster than males, the functional significance of which is not yet clear (Shah et al., 2020). These tantalizing findings suggest that there is much to be learned about glial sex‐specific differences, and we are excited for modern ‐omics approaches to help accelerate this understudied area of biology.

Another area we look to with anticipation is the study of glia in the context of aging and neurodegeneration. Sc/nRNA‐seq datasets of aged animals suggest class‐specific changes in glia upon aging (Davie et al., 2018; Lu et al., 2023). When considering four different “aging features,” cell composition, differentially expressed genes, number of expressed genes and cell identity decline (marker gene expression in young vs. aged animals), glial clusters were particularly variable, with astrocytes being the most age‐altered glia, while perineurial glia were the least age‐altered (Lu et al., 2023). Comparing these datasets with those of disease models of neurodegeneration (e.g., Alzheimer's disease model) (Wu et al., 2023) could provide needed insight into the timelines of glial versus neuronal dysfunction and in turn into glial contributions to neurodegeneration as well as potential glial interventions.

### 6.2 Caenorhabditis elegans

While rapid strides have been made, study of *C. elegans* glia is still in early stages. The glial molecular atlas by snRNA‐seq in this animal is a powerful addition to its pan‐neuronal scRNA‐seq atlas, and mapped connectome across sexes (Cook et al., 2019; Purice et al., 2023; Taylor et al., 2021; White et al., 1986). Importantly, it has enabled annotation of glia across sexes. These global molecular and cellular maps yield unprecedented insight into the nervous system of a multicellular animal. As more bulk, sc/nRNA‐seq whole‐animal datasets are rapidly being generated across animal life stages, physiological contexts, and environmental perturbations (Cao et al., 2017; Ghaddar et al., 2023; Packer et al., 2019; Purice et al., 2023; Roux et al., 2023; Taylor et al., 2021), it will be interesting to re/annotate glial cells in whole‐animal datasets based on the glia atlas and other studies (Bacaj et al., 2008; Fung et al., 2020; Grant et al., 2015; Katz et al., 2019; Purice et al., 2023; Stefanakis et al., 2024). For this, additional glia‐focused and bulk cell‐specific RNA‐seq studies will be informative, as will the extension of iterative *in silico* and in vivo approaches to identify glial clusters accurately. The invariant anatomy, mapped connectome, well‐studied neurons and neural circuits, and simple behaviors, provide an unparalleled platform for seamless focus between single glia–neuron and mechanistic studies to global circuit analyses. We suggest that such studies in this animal will offer enormous power to rapidly define how dynamic molecular changes in glia impact neural development, aging, sex, or disease states at single‐cell resolution. The molecular conservation observed from granular mechanistic studies, as well as insights derived from molecular profiling, suggests that these studies will shed light on evolutionarily conserved modules of glial functions that are relevant across species. Moreover, this optically transparent animal is amenable to in vivo calcium imaging in freely behaving animals (Atanas et al., 2023). Thus, ‐omic profiling of glia can readily lead to overlay of specific molecular perturbation to circuit dynamics. Finally, as multiomics at single‐cell and with spatial resolution will continue advancing via greater sensitivity, improvements in throughput, and reduction of cost, we expect future of *C. elegans* glia ‐omics research will include proteomics, metabolomic and measurements of the epigenome, all of which are currently unexplored avenues of inquiry. We also envision the extension of such approaches to study the molecular contribution of glia to disease in established *C. elegans* models of disease.

### 6.3 Cross‐species synergy

Comparative bioinformatics analysis of glial cells between model organisms like *C. elegans* and *Drosophila* has yet to be explored. Developing computational tools to do so will be invaluable. Such molecular comparisons could highlight evolutionarily conserved similarities in glial function, gene expression patterns, and regulatory networks. Recent functional and granular studies of glia in both *C. elegans* and *Drosophila* (Lago‐Baldaia et al., 2020; Singhvi et al., 2024) underscore the potential for such an approach, as well as functional conservation with vertebrate glia functions in health, disease, and aging.

## AUTHOR CONTRIBUTIONS

All authors contributed to the planning, writing, and revision of the manuscript. MDP and IL‐B contributed equally.

## Data Availability

Data sharing not applicable to this article as no datasets were generated or analysed during the current study.
